# Direct Reduction of Graphene Oxide/Nanofibrillated Cellulose Composite Film and its Electrical Conductivity Research

**DOI:** 10.1038/s41598-020-59918-z

**Published:** 2020-02-20

**Authors:** Junjun Chen, Hailong Li, Lihui Zhang, Chao Du, Tao Fang, Jian Hu

**Affiliations:** School of light industry and engineering, South China University of Technology, Guangzhou, 510641 China

**Keywords:** Electronic properties and devices, Electronic properties and materials

## Abstract

With the rapid development of wearable and portable electronic devices, it is increasingly important to develop conductive paper-like films (CPFs) with the characteristics of light, thin and self-supporting. In this paper, nanofibrillated cellulose (NFC) was used as reinforcing phase of film-forming to combine with graphene oxide (GO). Then graphene-based CPFs were prepared by directly reducing the GO/NFC composite film without any additional adhesives, which effectively avoided the difficulties of dispersion and combination with other materials caused by direct using of high content graphene. Meanwhile, three representative reduction methods for direct reduction of GO/NFC composite films were also compared. The results show that 450 °C thermal reduction and hydroiodic acid reduction were more effective than ascorbic acid reduction. On this basis, hydroiodic acid reduction and thermal reduction were used to discuss the effect of NFC addition to the conductivity of the film. This occured when increasing the content of NFC from 10% to 50%, the electrical conductivity of the composite film by hydroiodic acid reduction decreased from 153.8 S/m to 22.2 S/m. While the conductivity of composite film increased first and then decreased after thermal reduction both at 450 °C and 550 °C. What’s more, when NFC content was about 16.6% the electrical conductivity reached a high level which was 86.21 S/m and 168.9 S/m, respectively. This study provides a groundwork for the further development of graphene-based CPFs with low square resistance and high conductivity in large-scale preparation.

## Introduction

In recent years, electronic technology has developed rapidly in the direction of intelligence, green, microminiaturization and integration. Accordingly, three-dimensional conductive papers (CPs) or conductive paper-like films (CPFs) with light, thin, self-supporting and excellent durability features exhibit attracting prospect for applications in wearable device^1,2^ and the flexible electronic device^3–6^.

Graphene is a two-dimensional carbon nanomaterial with carbon atoms formed by sp^2^ hybridization, which has attracted ever-increasing attention by researchers for its excellent electrical, thermal and mechanical properties^7^. And it also has been widely researched in the preparation of conductive materials by virtue of its high electron mobility and high conductivity^8^. El-Kady^9^ *et al*. used LightScribe DVD optical drive to do the direct laser reduction of graphite oxide (GO) films to graphene with high electrical conductivity (1738 S/m), but the method requires high equipment and the laser-scribed process is difficult to control in actual production. Zhang^10^ *et al*. prepared highly conductive graphene nanomesh films (27800–35400 S/m) by an *in-situ* carbothermal reduction strategy and well performed in supercapacitors. However, the preparation process is relatively complicated and the efficiency needs to be improved in large-scale preparation. Among various approaches to prepare graphene composite CPs or CPFs, the development of simple, low-cost, large-scale, commercial methods is still a challenge. Kang^11^ *et al*. used traditional papermaking methods to prepare CPFs with conductivity of 11.6 S/m and square resistance of 1063 Ω/sq by mixing pulp with graphene. It exhibited high electrochemical performances in flexible electrodes for supercapacitors and lithium batteries. While Luong^12^ *et al*. utilized hydrazine to reduce the mixture of graphene oxide(GO) and nanocellulose to prepare conductive film by filtering, when the graphene content was 10%, the composite film conductivity was 71.8 S/m. Besides, simple solution process such as dipping^13,14^, coating^15^ and casting^16^ were also studied by researchers. However, in the above report, graphene was combined with other materials at a lower content, the conductivity of the materials was limited which may affect the property of the final device. To fully demonstrate the prominent electrical property of graphene and further improve the electrical conductivity of composites, increasing the proportion of graphene or using graphene as the main component to prepare CPFs is concerned. But in this process, two problems are easy to occur: First, graphene agglomerate easily and disperse in poor state, it may cause local graphitization and affect its performance. Second, the surface inertness of graphene makes it difficult to form a film itself, and it’s also hard to combine well with other materials. So the preparation of graphene-based CPFs and the improvement of its conductive properties need to be further studied.

GO as an oxide of graphene, has a large number of oxygen-containing groups on its surface and edge, showing good dispersion in water. More importantly, it can be reduced into graphene to restore its conductivity^17^. While nanofibrillated cellulose (NFC) not only has large specific surface area, high strength, good biodegradability, but also larger in length-diameter ratio which can easily interweave a network structure^18^. Besides, NFC has high hydrophilicity due to its large amount of hydroxyl groups, and is easy to form films or gelations^19^. Therefore, in this paper, NFC was used as the enhancement phase of film formation. By adding an appropriate amount of NFC to combine with well dispersed GO into film, the graphene-based CPFs were obtained by direct reduction of the GO/NFC composite film, thus it effectively avoided the difficulties of dispersion and combination with other materials caused by direct using of high content graphene.

Furthermore, different reduction methods are of notable effect on the conductivity of the reduced graphene oxide(RGO), which accordingly influence the conductivity of GO/NFC composite film after reduction. Commonly used methods for reducing GO are mainly divided into chemical reduction method and thermal reduction method. The high-efficiency reducing agents used in chemical reduction ordinarily includes hydrazine and its derivatives^20–22^, sodium borohydride^23^, hydroiodic acid (HI)^24,25^ and green reducing agents such as ascorbic acid (VC)^26^, chitosan^27^ and glucose^28^. As an efficient, non-toxic reducing agent, HI can directly reduce GO film into high conductive graphene film without destroying the integrity and flexibility of the film^24^. When VC is used as reducing agent, RGO obtained after reduction has a good dispersibility, which is beneficial to the further application of the product^26^. In addition to the chemical reduction method, thermal reduction^29–31^ is also an efficient reduction method. The mechanism is that the oxygen-containing functional groups on GO escape from the sheet layer in the form of H_2_O, CO_2_ or CO at a high temperature by using GO under the protection of inert gas, then RGO can be obtained.

Hence, HI reduction, VC reduction and thermal reduction were investigated to prepare graphene-based CPFs by direct reduction of the GO/NFC composite film. The effects of different reduction methods on the surface properties, microstructure and electrical conductivity of the composite film before and after reduction were discussed. On this basis, we also discussed the effect of NFC addition to the conductivity of the graphene-based CPFs, and analysed reasons for the change in conductivity.

## Experimental

### Reagents and instruments

Bleached eucalyptus pulp, Graphene oxide dispersion (Beijing carbon Century Science and Technology Co., Ltd., 300 mesh). HI (Aladdin Reagents Co., Ltd., 55–58%), VC, TEMPO, NaBr, NaClO, NaOH (All chemicals were of analytical grade). Deionized water was used in this study. Vacuum tube furnace TF1200–200 (Kunshan Acson Machinery Co., Ltd.).

### Preparation of NFC

Eucalyptus pulp is first oxidized by TEMPO^32^, and washed with HCl (1 mol/L) three times, then homogenized by high pressure to obtain a gel-like NFC.

### Preparation of GO/NFC composite film

The above-mentioned prepared NFC and a certain concentration of GO were mixed by ultrasound. The mixed solution with different mass ratio of GO and NFC was prepared respectively (absolute dry mass ratio). Then, filtration of mixed solution into film (quantitative 19.8 g/m^2^). After drying at room temperature (30 °C) for 24 h, the composite film was retained in the following reduction experiments.

### Reduction of GO/NFC composite film

#### HI reduction

The GO/NFC composite film was immersed in a sealed container containing (55–58%) HI solution and reduced it in a constant temperature water bath at 90 °C for 1 hour. After that, the residual solution on the film was washed out by ethanol and dried naturally at room temperature

#### VC reduction

The GO/NFC composite film was immersed in a sealed container containing VC solution (the mass of VC is 10 times than that of GO), which was reduced in a constant temperature water bath at 90 °C for 1 hour. After that, the residual solution on the film was washed out by ethanol and dried naturally at room temperature.

#### Thermal reduction

The GO/NFC composite film is placed in a vacuum tube furnace under the protection of nitrogen, the film was heated to 450 °C for 0.5 h, then dropped to room temperature.

### Characterizations

The surface morphology and microstructure of the samples were analyzed by Field Emission Scanning Electron Microscopy(FESEM) (Merline, Zeiss). X-ray diffraction(XRD) patterns were recorded on a Bruker D8 advance X-ray diffractometer using Cu Kα radiation (λ = 0.15406 nm). Fourier transform infrared spectroscopy(FTIR) (vertex70, bruker) with wave range of 4000^−1^–400 cm^−1^ was used to identify and analyze the structure and composition of the samples. Microscopic Raman spectroscopy (LabRAM Aramis, Horiba Jobin Yvon) was used to analyze the microstructure changes of the samples. X-ray photoelectron spectroscopy (XPS) was used to further study the evolution of functional groups. Four-probe (RTS-9, Guangzhou four-probe technology) was used to test the square resistance and conductivity of the sample. Multiple measurements and the average values were obtained. Tensile strength (TS), Young’s modulus (YM) and the stress-strain curves of GO films and GO/NFC composite films after different reduction methods were conducted by using the Universal Testing Machine (INSTRON 5565, Norwood, MA, USA) at a speed of 10 mm min^−1^ with films 1.5 cm wide and 3 cm long.

## Results and Discussion

**Figure 1:**
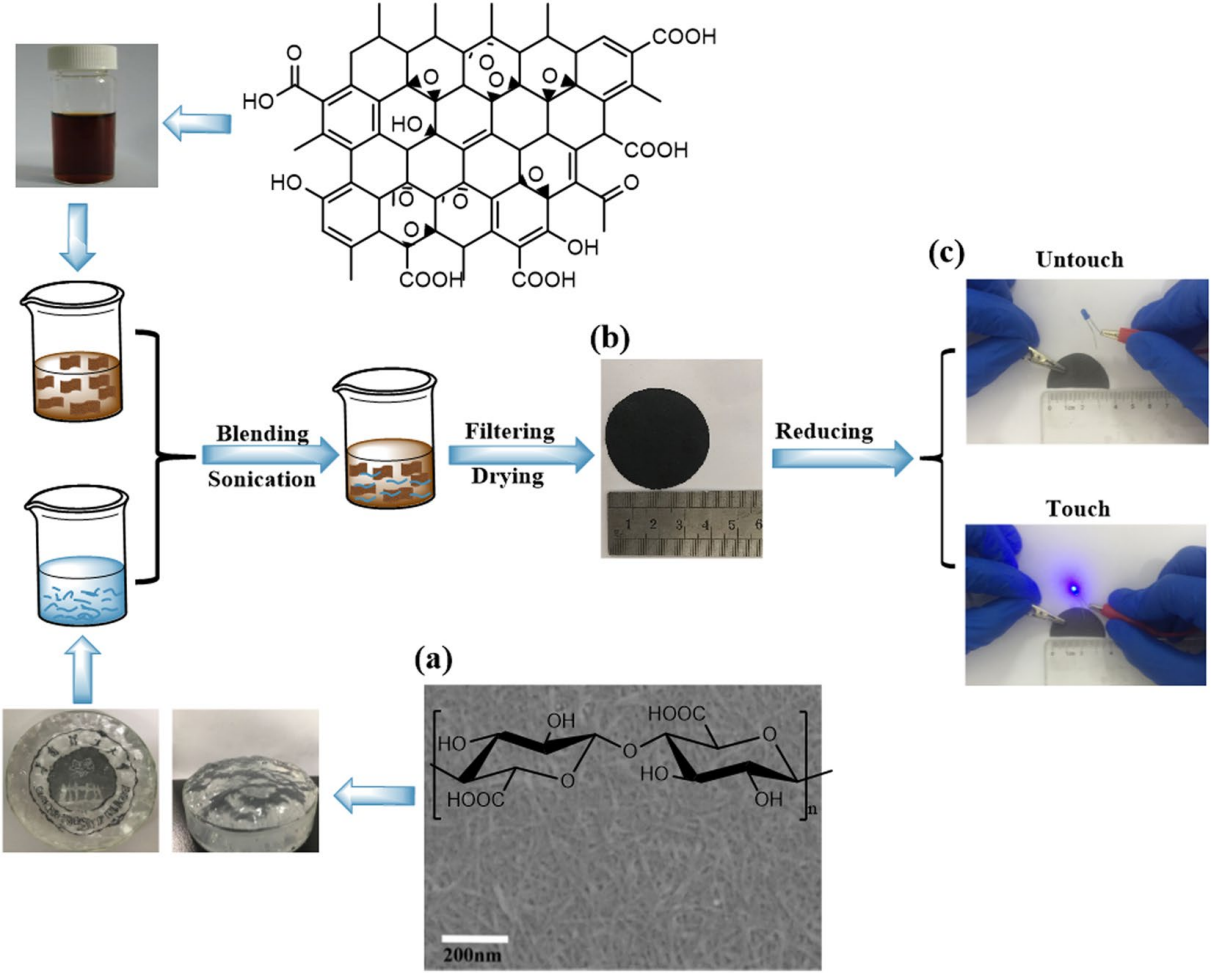
Illustrates the fabrication process of the graphene-based CPFs. (**a**) Prepared NFC SEM image; (**b**) Photographs of GO/NFC composite film; (**c**) The photograph of graphene-based CPFs.

Figure 1 illustrates the fabrication process of the graphene-based CPFs. Prepared NFCs were uniform with a width of 5–10 nm, and high aspect ratio (Fig. 1a). GO and NFC were mixed by ultrasound and then filtered in a certain proportion to obtain GO/NFC composite films (Fig. 1b), which combined well under the action of hydrogen bonds. Then, graphene-based CPFs were prepared by direct reduction of GO/NFC composite films, which could make the LED lamp glow steadily (Fig. 1c).

### The effect of different reduction methods on the graphene-based CPFs

**Figure 2:**
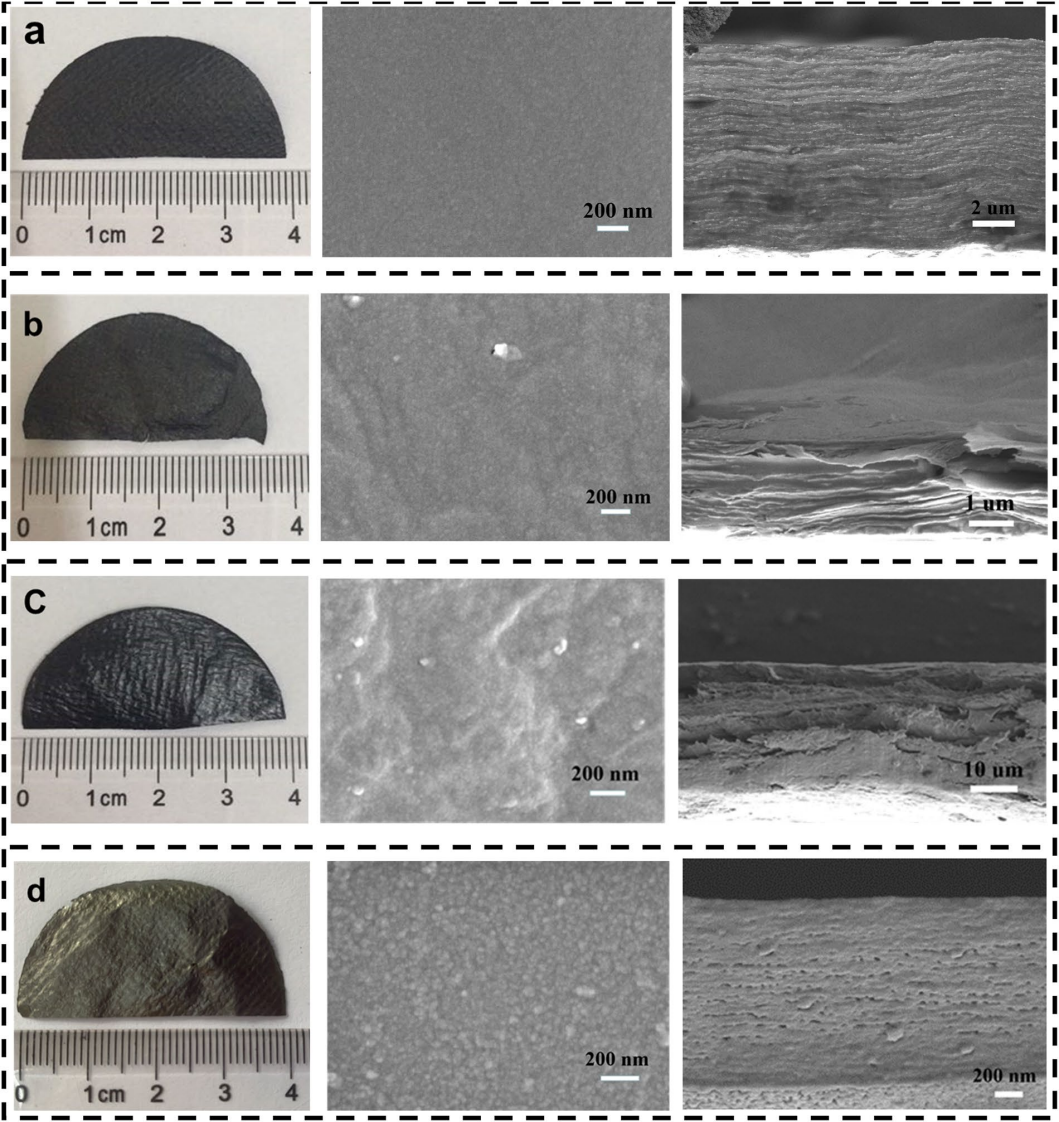
Illustrates the fabrication process of the graphene-based CPFs. (**a**) Prepared NFC SEM image; (**b**) Photographs of GO/NFC composite film; (**c**) The photograph of graphene-based CPFs.

Figure 2 shows photographs and SEM images of the GO/NFC composite film before and after reduction (GO: NFC = 1:1). In Fig. 2a, surface of GO/NFC film was smooth and the lamellar structure was compact under the condition of vacuum filtration, which due to hydrogen bonds between GO and NFC. Moreover, thickness of GO/NFC composite films were decreased after reduction and it also kept well in shape, according to Fig. 2b–d. After reduction process, the compact layer-like structure after HI reduction was warped, and orderly layered structure after VC reduction was destroyed, there were also many small holes in the cross-section after thermal reduction (450 °C), which can be observed in the surface and cross section of SEM images (Fig. 2b–d). All of these phenomena were related to the disappearance of hydrogen bonds and the lattice shrinkage caused by the recovery of sp^2^ hybridization when oxygen-containing functional groups were removed.

Next, the square resistance and conductivity of graphene-based CPFs were measured, which was shown in Table 1. The electrical conductivity of composite films after 450 °C thermal reduction and HI reduction is obviously better than that of VC reduction. Although the conductivity of the composite film after HI reduction is close to thermal reduction, its square resistance is relatively small. Owing to the fact that the intense carbonization of NFC during thermal reduction, resulting in a smaller thickness of the composite film.

**Table 1:** The conductive properties of graphene-based CPFs.

Reduction Method	Film Thickness (mm)	Square Resistance (Ω/sq)	Conductivity (S/m)
VC Reduction	0.12	10^4^	0.83
HI Reduction	0.1	450	22.22
Thermal Reduction (450 °C)	0.05	854	23.42

The relationship between sheet resistance and resistivity is as follows:

1 $${\rm{R}}={\rm{\rho }}/{\rm{d}}$$

ρ is the resistivity of the material, d is the thickness. When the resistivity is closely, the square resistance is inversely proportional to the thickness, so the square resistance of the thermal reduction is larger.

To gain further insight into the level of the reduction effects of VC reduction, HI reduction and thermal reduction, Raman spectroscopy, XRD, FTIR, XPS were employed to investigate the structural evolution during the reduction process.

**Figure 3:**
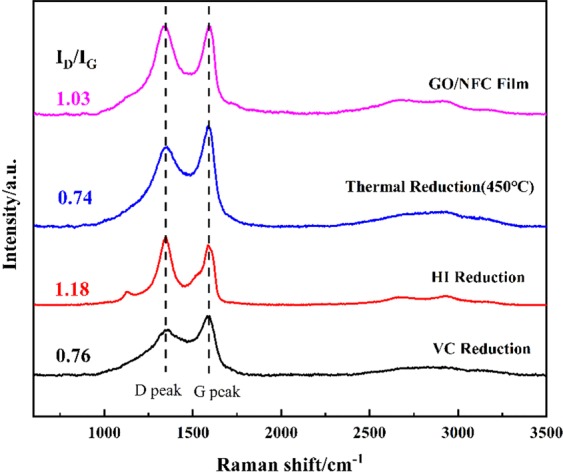
Raman spectra of GO/NFC composite film (GO: NFCs = 1:1) before and after reduction.

Raman spectroscopy is an effective method to characterize the structural changes of defects and edge groups in carbon materials. The intensity ratio of D peak at 1350 cm^−1^ and G peak at 1575 cm^−1^ in Raman spectrum is often used to evaluate the sp^2^ hybrid domain size of carbon materials. What the sp^3^/sp^2^ carbon atom ratio can indirectly reflect the degree of disorder in the structure^33^. Figure 3 shows the Raman spectrum before and after reduction of GO/NFC composite film. During the reduction process, a new hybrid region of sp^2^ would be formed, which decreased the ratio of I_D_/I_G_ accordingly. By comparing the ratio of I_D_/I_G_, 450 °C thermal reduction (0.744) is intense than VC reduction (0.762) due to the formation of larger sp^2^ hybrid region at high temperature. While the I_D_/I_G_ ratio of the composite film after HI reduction (1.118) is higher than that of the GO/NFC composite film (1.033), the reason for this is that the removal of oxygen atoms in the reduction process of GO was too intense and it inevitably led to disorder. Although the size of the new hybrid domain is smaller than that of GO, the number of new hybrid domains is larger, which brings about the increasing of I_D_/I_G_ ratio^34,35^.

**Figure 4:**
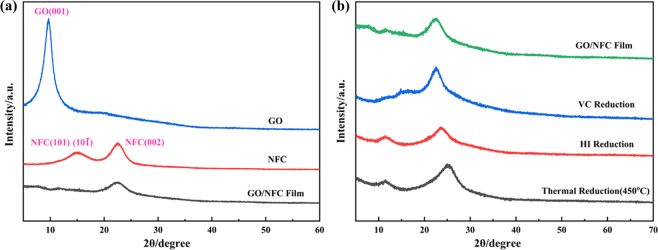
(**a**) XRD spectra of GO, NFC, GO/NFC composite film (GO: NFCs = 1:1). (**b**) XRD spectra of GO/NFC composite film (GO: NFCs = 1:1) before and after reduction.

In addition, the changes of crystal structure before and after reduction of GO/NFC composite films were also characterized by XRD. Figure 4(a) shows the XRD patterns of GO, NFC and GO/NFC composite film (GO: NFC = 1:1). NFC exhibited a distinct peak at 2θ = 22.6°, corresponding to the (002) lattice plane of cellulose I, and two overlapping diffraction peak at about 14.0°–17.8° correspond to the (101) and (10$\bar{1}$) lattice faces of the cellulose I crystal structure^36,37^. While 2θ = 10.8° corresponds to the characteristic peak of GO (001) lattice plane^38^. Additionally, the GO/NFC composite film exhibited the (002) crystal plane of NFC at 2θ = 22.6°, while the characteristic peak of the GO (001) crystal plane and the overlapping diffraction of NFC nearly covered, which indicates the good combination of GO and NFC.

Figure 4(b) is the XRD patterns of GO/NFC films (GO: NFC = 1:1) before and after reduction. The XRD patterns of the composite films obtained by three reduction methods did not show a peak at 10.8°, suggesting that graphene oxide was reduced. Moreover, the diffraction peak of the reduced composite film at 2θ = 24.8° is close to the diffraction peak of graphite (002) lattice planes^39^. When the GO/NFC composite film respectively reduced by VC reduction, HI reduction and thermal reduction, the characteristic peak of 24.8° shifted to large angle in turn. From the formula 2dsinθ = nλ, with the increase of θ angle, the corresponding crystal-plane spacing d decreases. The removal of oxygen-containing functional groups in different reduction processes would affect the spacing between lattice planes, which indicated that the composite film was closer to the crystal structure of graphene after thermal reduction. Meanwhile, the conclusion that new hybrid regions of sp^2^ were formed and the ratio of ID/IG decreased in Raman spectra was also verified.

**Figure 5:**
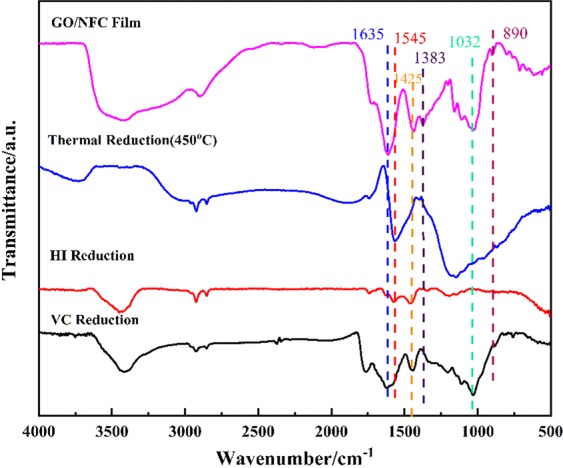
FTIR spectra of GO/NFC composite film (GO: NFCs = 1:1) before and after reduction.

The change of functional groups of GO/NFC composite films (GO: NFC = 1:1) before and after reduction were characterized by FTIR. As shown in Fig. 5, the oxygen-containing groups were removed in different degree after the three reduction methods: the in-plane O-H bending vibration absorption peak (1383 cm^−1^) in the NFC and GO, C-O-C stretching vibration peak (890 cm^−1^), C-OH stretching vibration peak (1032 cm^−1^) and COOH stretching vibration peak (1635 cm^−1^) in GO (identification similar to that reported^40–42^). Typically, intensity of peak at 1425 cm^−1^ belongs to the bending vibration absorption peak of CH_2_ in NFC^43^, which was removed only after thermal reduction. This suggests that the carbonation of NFC during thermal reduction. At the same time, the strong absorption peak at 1543 cm^−1^in thermal reduction and HI reduction can be clearly observed, which caused by the stretching vibration of sp^2^ chemical bond in the graphene structure^42^. The results show that the sp^2^ electron conjugate structure of graphene can be reconstructed effectively after reduction.

**Figure 6:**
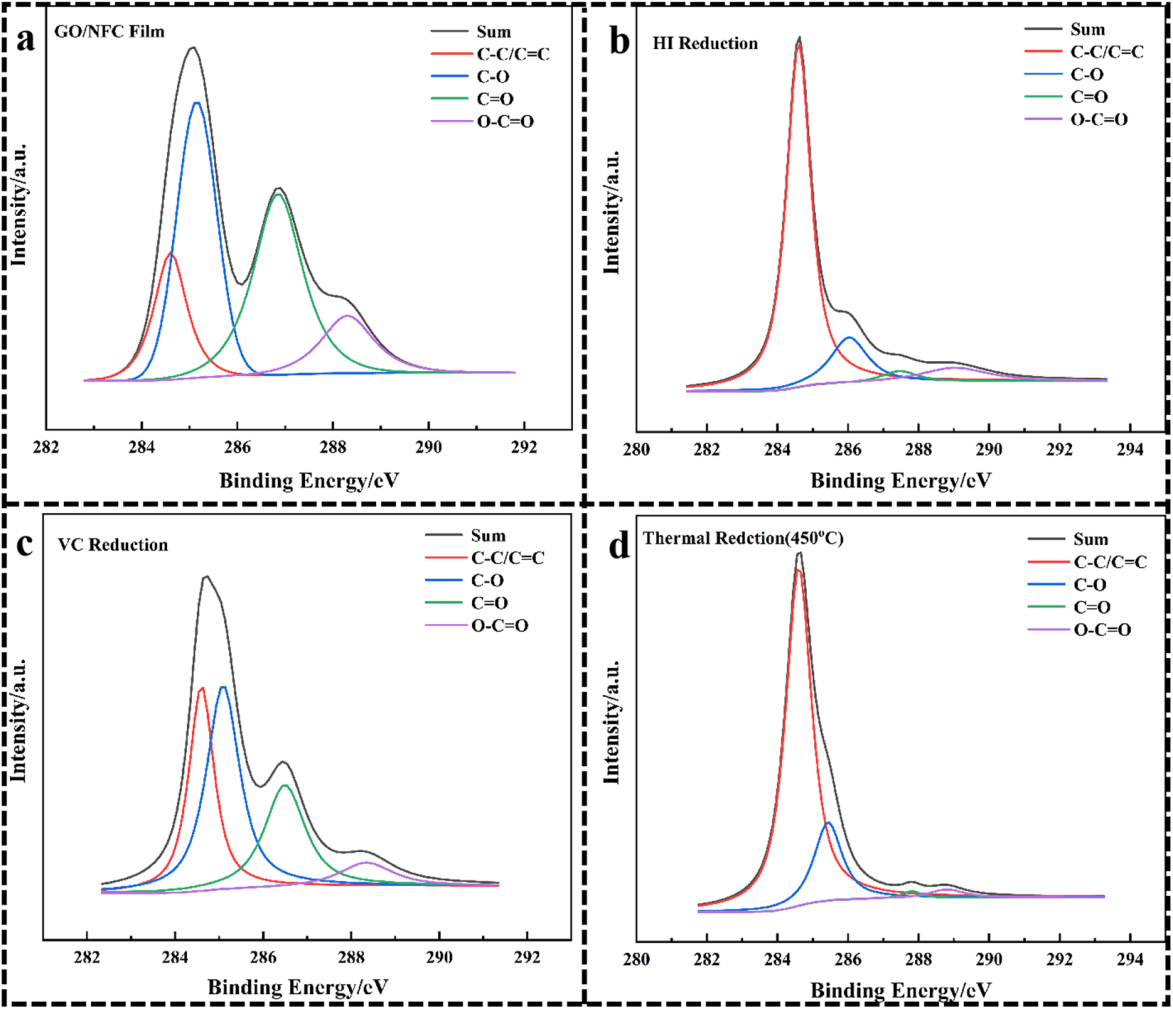
High-resolution XPS C1s spectra of GO/NFC composite film (GO: NFCs = 1:1) before and after reduction.

The evolution of functional groups in GO/NFC composite films (GO: CNFs = 1:1) surface before and after reduction were further determined by XPS. After reduction, the C/O atom ratio was increased accordingly in Table 2, which also confirms the removal of oxygen in the film during reduction. It’s apparent that the deoxidation of thermal reduction was the strongest, which can be seen from the increase of C/O atomic ratio from 2.59 to 8.04. Moreover, in Fig. 6 high-resolution XPS C1s spectrum of GO/NFC composite film before and after reduction clearly indicated the presence of four types of carbon atoms in different functional groups: the unoxygenated ring carbon C-C/C=C(~284.4 eV), epoxy carbon, hydroxyl carbon and ether carbon C-O(~286.4 eV), carbonyl carbon C=O(~287.8 eV), carboxylate carbon O-C=O(288.8 eV), which is similar to other reports^37,40^. This indicates the removal of epoxyl, carboxyl, and carbonyl functional groups, and a simultaneous increase in graphitic carbon after reduction.

**Table 2:** Contents of the surface elements of GO/NFC composite films (GO: NFCs = 1:1) before and after reduction.

Samples	C At%	O At%	C/O
GO/NFC Film	72.13	27.87	2.59
VC Reduction	78.60	21.40	3.67
HI Reduction	86.06	13.94	6.17
Thermal reduction (450 °C)	88.94	11.06	8.04

**Figure 7:**
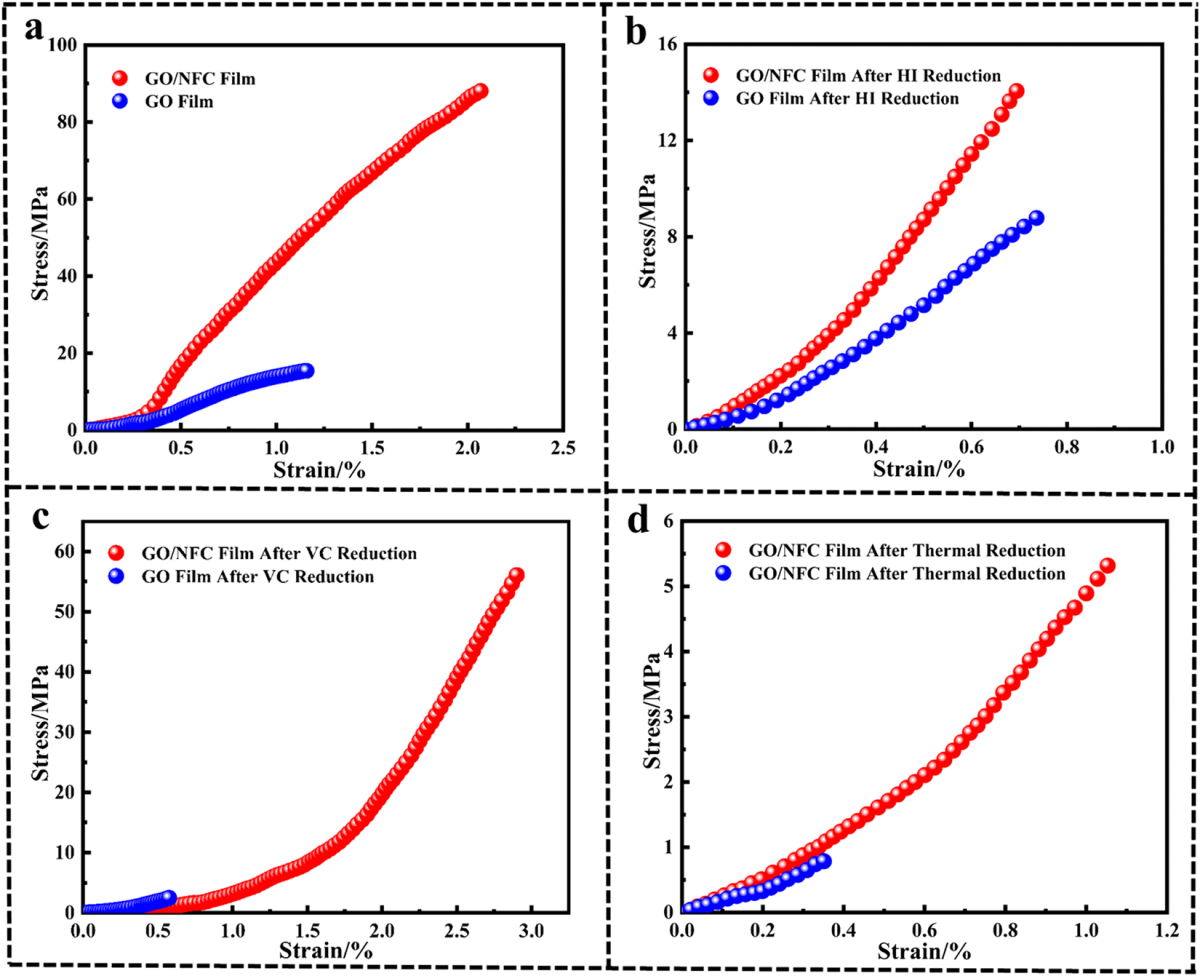
Stress-strain curves of GO films and GO/NFC composite films (GO: NFC = 1:1) before and after reduction.

Furthermore, stress-strain curves of GO film and GO/NFC composite film (GO: NFC = 1:1) before and after reduction were shown in Fig. 7. Overall, the addition of NFC can enhance the strength of GO/NFC composite film (GO: NFC = 1:1) before and after reduction, which can be clearly observed from TS and YM listed in Table 3. Compared with GO film, the results showed that the TS of GO/NFC composite film increased from 15.41 MPa to 88.06 MPa, the YM increased from 2.14 GPa to 5.56 GPa. Meanwhile, the addition of NFC can also increase the strength of composite films after reduction (Fig. 7b–d) compared with RGO films. It can be clearly obtained from Table 3 that the TS of the GO/NFC composite films were 1.6 times, 23.5 times and 6.7 times than that of the GO films after HI reduction, VC reduction and thermal reduction. Similarly, the YM of the GO/NFC composite films were also enhanced 1.8 times, 2.4 times and 5.7 times than that of the GO films after HI reduction, VC reduction and thermal reduction.

**Table 3:** Tensile strength (TS) and Young’s modulus (YM) of GO films and GO/NFC composite films (GO: NFC = 1:1) before and after reduction.

Sample	YM (GPa)	TS (MPa)
GO Film	2.14	15.41
GO Film-HI Reduction	1.57	8.78
GO Film-VC Reduction	0.77	2.49
GO Film- Thermal reduction	0.33	0.79
GO/NFC Film	5.56	88.06
GO/NFC Film-HI Reduction	2.90	14.05
GO/NFC Film-VC Reduction	4.33	56.06
GO/NFC Film-Thermal reduction	0.80	5.32

Nevertheless, due to the disappearance of hydrogen bonds caused by the remove of oxygen-containing functional groups, the compact lamellar structure is also damaged in varying degrees, which is approved by the description of SEM results (Fig. 2b–d), so the strength of composite films after reduction would be reduced in varying degrees (Fig. 7b–d).

### The effect of NFC addition on conductivity of the graphene-based CPFs

Because the conductivity of the composite film after HI reduction and thermal reduction were much better than that of VC reduction. Thus, the effect that addition of NFC in the conductivity of the graphene-based CPFs was discussed below.

**Figure 8:**
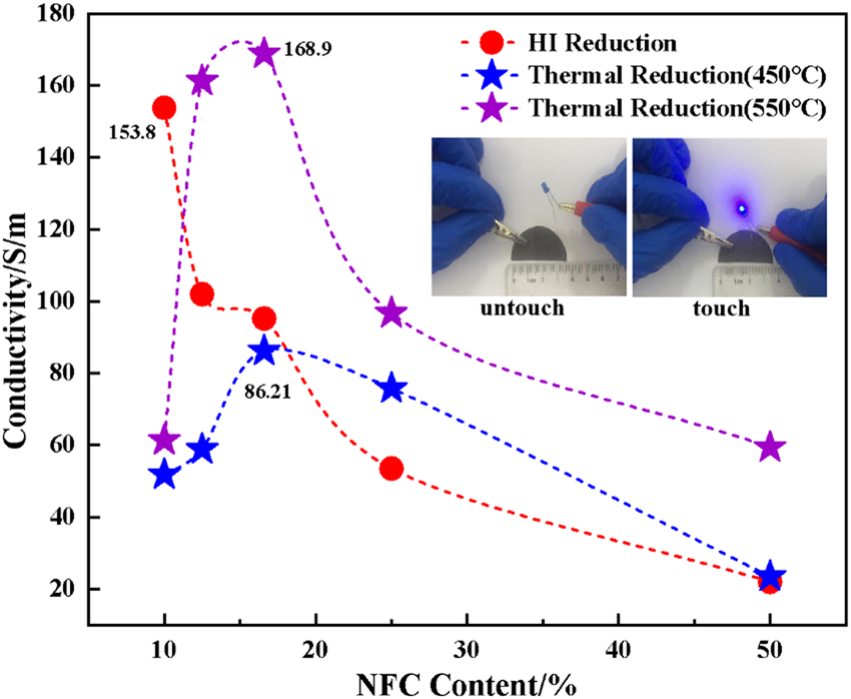
The conductivity of the graphene-based CPFs respectively prepared by thermal reduction and HI reduction with different NFC content.

Figure 8 shows the conductivity of GO/NFC composite films with different NFC contents after HI reduction and thermal reduction (450 °C, 550 °C) respectively. This occured when increasing the content of NFC from 10% to 50%, the electrical conductivity of the composite film by HI reduction reduced from 153.8 S/m to 22.2 S/m. It is obvious that the electrical conductivity was improved by the gradual reduction of GO into graphene during the reduction process. Thus, the conductivity of the film decreased after reduction due to the decrease of GO. Besides, the aromatization of NFC can also provide partial conductivity at high temperature. Compared with 450 °C thermal reduction, the conductivity is higher at 550 °C in the same dosage of the NFC. This due to the fact that a higher temperature would bring the greater degree of aromatization and removal of oxygen-containing functional groups.

Meanwhile, different from HI reduction, the conductivity of the film after thermal reduction increased first and then decreased both at 450 °C and 550 °C. When NFC content was about 16.6%, the electrical conductivity reached a high level which were 86.21 S/m and 168.9 S/m, respectively. That could be the composite film was well intertwined and formed a good conductive network structure.

Both of these are beneficial to the electron transport after reduction and make the composite film achieve the best conductivity. When the dosage was less than 16.6%, NFC was not enough to form a uniform net conductive structure. What is the reason for the decrease of conductivity after the reduction is that the graphene was easily deposited and covered which could bring the thermal reduction reaction was not thorough and the carbonization of the NFC was also suppressed. Then, we took the film after HI reduction as a sample (NFC accounts for 12.5%) to do diode path experiment. When the LED lamp touched with the conductive film prepared above, the LED lamp can emit light stably. This indicates that prepared graphene-based CPFs possess excellent conductivity.

## Conclusions

In this paper, the graphene-based CPFs were successfully prepared by directly reducing the GO/NFC composite film without any additional adhesives, which effectively avoided the difficulties of dispersion and combination with other materials caused by direct using of high content graphene. Moreover, the surface characteristics, microstructure and electrical conductivity of composite films after HI reduction, VC reduction and thermal reduction were compared and analyzed. The results show that thermal reduction and HI reduction were more efficient than VC reduction, and NFC would be carbonized at the same time during thermal reduction. On this basis, we also discussed the effect of NFC addition to the conductivity of graphene-based CPFs prepared by HI reduction and thermal reduction. With the increase of NFC content from 10% to 50%, the conductivity of the composite film decreased gradually after HI reduction. In comparison, the conductivity of the film increase first and then decrease after thermal reduction both at 450 °C and 550 °C. When NFC content was about 16.6%, the electrical conductivity reached a high level, which were 86.21 S/m and 168.9 S/m, respectively. This work provides a basis for the further development of flexible conductive film with low square resistance and high conductivity, and contributes to its potential applications in portable and wearable electronic devices.

## Supplementary information

Supporting information Direct Reduction of Graphene Oxide/Nanofibrillated Cellulose Composite Film and its Electrical Conductivity Research.
