# Supplementary material for: Direct Reduction of Graphene Oxide/Nanofibrillated Cellulose Composite Film and its Electrical Conductivity Research
Source: Sci Rep. 2020 Feb 20;10:3124. doi: 10.1038/s41598-020-59918-z (PMC7033249; doi:10.1038/s41598-020-59918-z)
Supplement: Supplementary file 1 — Supporting information Direct Reduction of Graphene Oxide/Nanofibrillated Cellulose Composite Film and its Electrical Conductivity Research. [file 41598_2020_59918_MOESM1_ESM.docx]

Supporting information

**Direct Reduction of Graphene Oxide/Nanofibrillated Cellulose Composite Film and its Electrical Conductivity Research**

**Junjun Chen** **^1^****, Hailong Li ^1^*, Lihui Zhang ^1^****, Chao Du** **^1^****, Tao Fang ^1^ and Jian Hu** **^1^**

^1^ School of light industry and engineering, South China University of Technology, Guangzhou 510641, China

***** Correspondence: [felhl@scut.edu.cn](mailto:felhl@scut.edu.cn)

Junjun Chen ORCID code: <https://orcid.org/0000-0002-7152-2848>


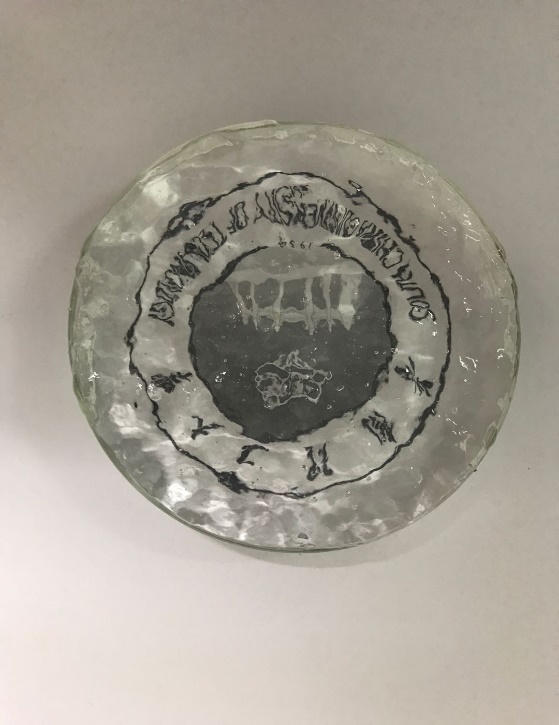

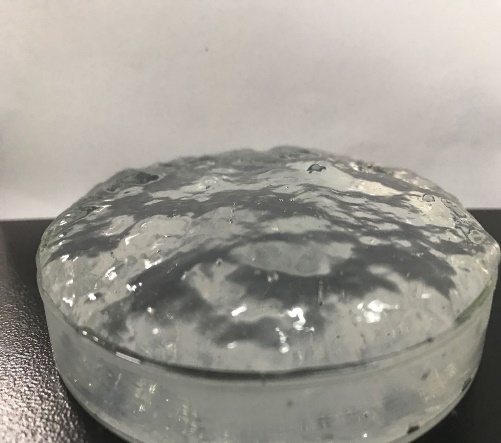


**Figure S1.** Photographs of the prepared NFC

**
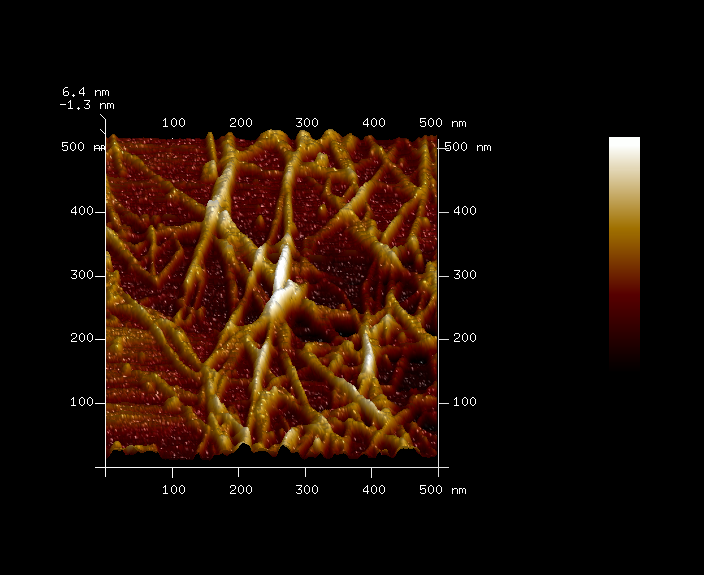
**

**Figure S2.** 3D AFM and SEM images of the prepared NFC


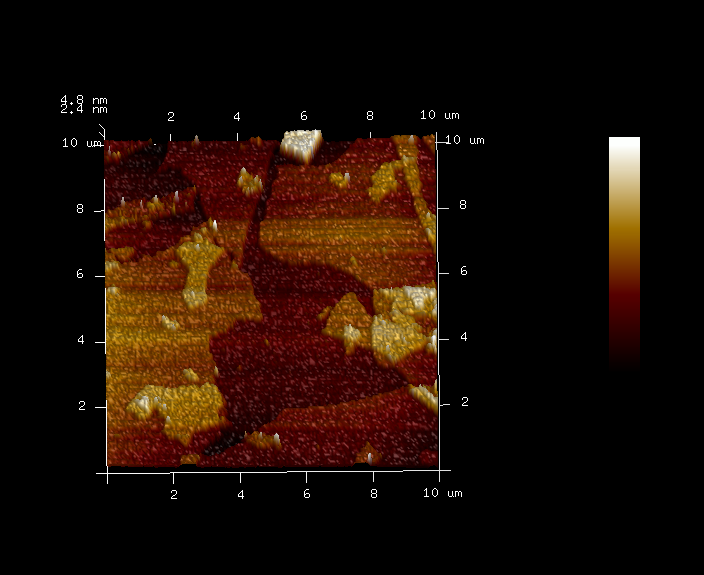


**Figure S3.** 3D AFM image of the prepared GO


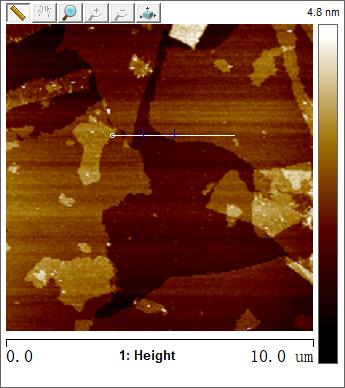


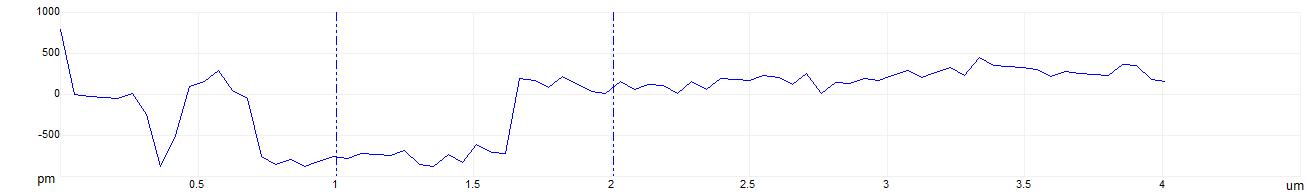


**Figure S4.** Graphene oxide sheet thickness measured by NanoScope Analysis 1.5
